# Identification and validation of biomarkers in gastric cancer-associated membranous nephropathy: Insights from comprehensive bioinformatics analysis and machine learning

**DOI:** 10.3389/fimmu.2025.1630836

**Published:** 2025-10-08

**Authors:** Qianqian Xu, Yue Yang, Cong Zhang, Min Tan, Jiayi Li, Wenge Li

**Affiliations:** ^1^ Department of Nephrology, China-Japan Friendship Hospital, Beijing, China; ^2^ Department of Nephrology, Peking University China-Japan Friendship School of Clinical Medicine, Beijing, China

**Keywords:** gastric cancer, membranous nephropathy, immunohistochemistry, bioinformatics analysis, machine learning

## Abstract

**Background:**

This study explores the genetic basis of membranous nephropathy (MN) in gastric adenocarcinoma (GC) through bioinformatics and machine learning analyses.

**Methods:**

Gene expression profiles from MN (GSE108109) and GC (GSE54129) datasets were obtained from the Gene Expression Omnibus. Common differentially expressed genes (DEGs) were identified using the limma R package. Biological functions were analyzed via Gene Ontology (GO) and Kyoto Encyclopedia of Genes and Genomes (KEGG) pathways with the Cluster Profiler package. LASSO regression and Random Forest algorithms were used to identify hub genes associated with GC-related MN. The area under the curve (AUC) of ROC analysis validated these genes for their diagnostic potential. Gene Set Enrichment Analysis (GSEA) and immune cell infiltration analysis were conducted, with hub genes validated through immunohistochemistry on renal and gastric cancer tissues.

**Results:**

We identified 40 common DEGs between GC and MN datasets. Using protein-protein interaction networks, 20 significant hub genes were selected, primarily involved in inflammatory and immune response regulation. Key hub genes identified were *CCND1*, *CEBPD*, *COL10A1*, and *BMP2*, which demonstrated high accuracy in discriminating MN. Notably, *CCND1*, *CEBPD*, and *BMP2* were significantly overexpressed in glomerular and gastric cancer tissues.

**Conclusions:**

Our findings highlight the crucial roles of *CCND1*, *CEBPD*, and *BMP2* in the pathogenesis of GC-associated MN, providing insights for future research and potential therapeutic strategies.

## 1 Introduction

Gastric cancer (GC) is one of the most prevalent cancers globally, accounting for approximately 4.9% of new cancer cases and 6.8% of cancer-related deaths annually, with a notably higher incidence in males (1). Among the various histological subtypes, gastric adenocarcinoma is the most common, representing about 90% of all gastric cancers (2). The multifactorial nature of GC, influenced by dietary factors, Helicobacter pylori infection, and genetic predispositions, underscores the complexity of its etiology and necessitates further exploration of associated systemic effects (3).

Among paraneoplastic glomerulopathies, membranous nephropathy (MN) is the most frequently reported and clinically significant subtype, accounting for a substantial proportion of malignancy-associated renal lesions (4). This makes MN particularly relevant when considering the systemic complications of GC. Since Lee’s seminal 1966 study postulated a link between nephrotic syndrome and malignancy, this association has gained increasing clinical significance, as nephrotic syndrome may herald an underlying malignancy and, conversely, treatment of the tumor can lead to remission of MN (5–7).

The pathophysiological link between cancer and MN is thought to involve immune complex deposition, tumor antigens that mimic podocyte proteins, and cross-reactive antibodies that trigger complement-mediated injury (8–10). Clinical observations that MN often improves following cancer therapy provide further support for these immune-mediated mechanisms (7). Although various glomerulopathies such as minimal change disease and focal segmental glomerulosclerosis have been reported in malignancy, MN is distinguished by its higher prevalence, stronger paraneoplastic association, and unique antigenic mechanisms, justifying its selection as the focus of the present study (11).

Recent advancements in high-throughput sequencing and bioinformatics have provided unprecedented opportunities to systematically investigate molecular mechanisms in complex diseases (9). Differentially expressed gene (DEG) analysis and machine learning algorithms such as LASSO and random forest can pinpoint hub genes with diagnostic and therapeutic potential (12).

Although previous studies have reported associations between malignancy and MN, the genetic and molecular mechanisms underlying gastric cancer–associated MN remain poorly understood. For the first time, we integrated multi-omics data and machine learning to identify GC-MN-specific hub genes and validate their cross-regulatory roles in GC cell proliferation and glomerular injury, complementing the established ‘molecular mimicry’ mechanism of cancer-associated MN (9).

## 2 Materials and methods

### 2.1 Data source

We searched the GEO database (https://www.ncbi.nlm.nih.gov/geo/) for membranous glomerulonephritis and gastric cancer (GC) data sets. Microarray data sets GSE108109 (44 Membranous Nephropathy and 6 Living donor) and GSE54129 (111 human gastric cancer tissues and 21 noncancerous gastric tissues) were downloaded from the GEO database.

**Figure 1:**
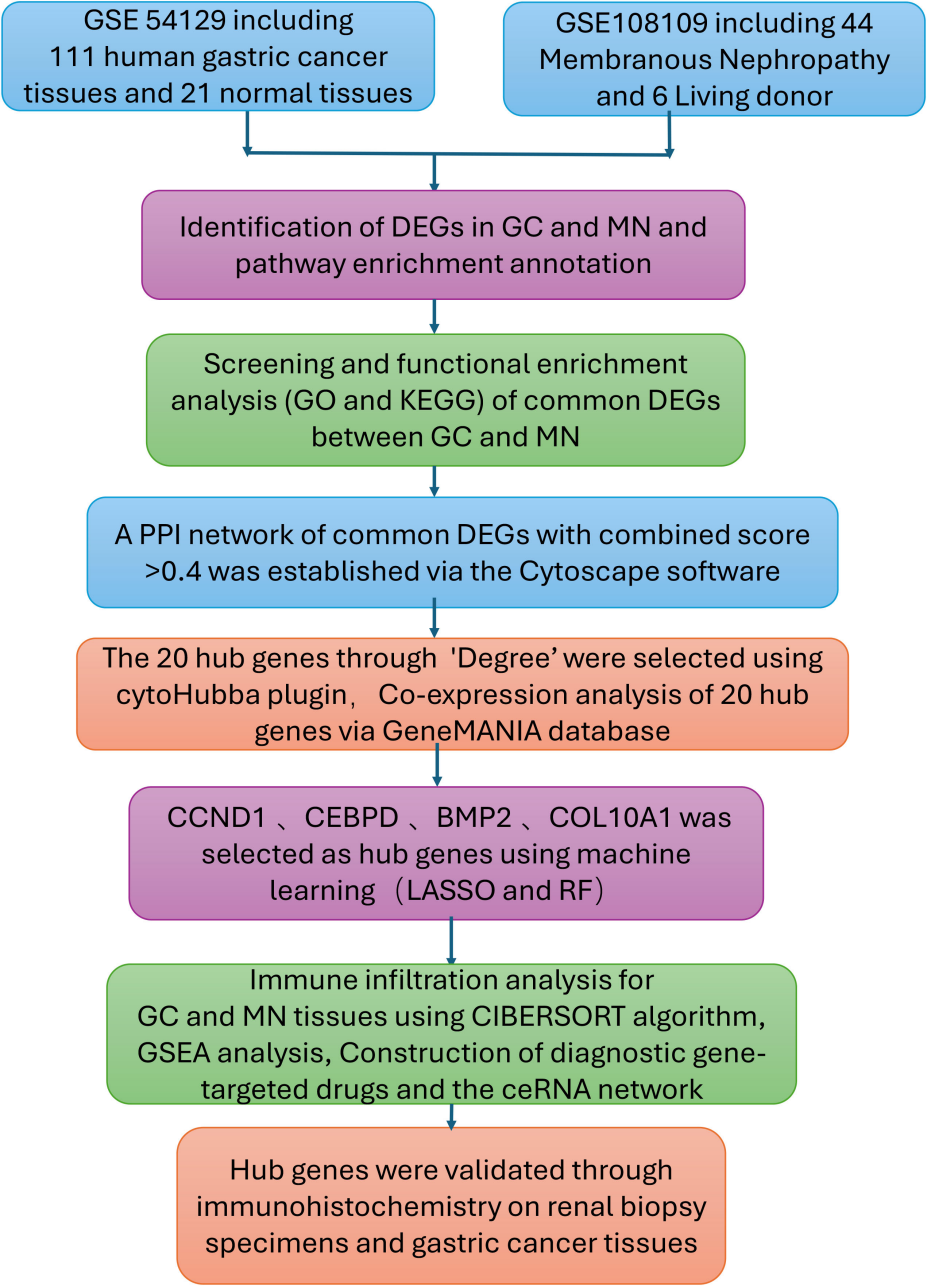
Research design flow chart.

**Figure 1** summarizes the work flow of data collection and analysis.

### 2.2 Analysis of differentially expressed genes

First, the normalizeBetweenArrays function from the limma R package (13) was employed to standardize gene expression measurements in both the membranous nephropathy (MN) dataset GSE108109 and the gastric cancer (GC) dataset GSE54129. Raw expression data from GEO datasets were normalized using the `normalizeBetweenArrays` function in the limma package, followed by log2 transformation to ensure comparability across samples. Batch effects between datasets were assessed using PCA, and probe-to-gene mapping was standardized prior to integration. Differentially expressed genes (DEGs) were defined using adjusted p < 0.05 and |log2 fold-change| ≥ 1, a threshold chosen to balance statistical rigor with biological interpretability and widely applied in transcriptomic studies. These DEGs were subsequently visualized through volcano plots and heatmaps using ggplot2 and heatmap in R. Finally, the VennDiagram package was used to pinpoint and depict overlapping DEGs shared by MN and GC.

### 2.3 Functional enrichment analysis of DEGs

To elucidate the biological mechanisms underlying the hub genes associated with both GC and MN, we conducted functional enrichment analyses. The Gene Ontology (GO) database offers comprehensive annotations for gene functions—covering molecular roles, biological pathways, and cellular components—while the Kyoto Encyclopedia of Genes and Genomes (KEGG) Pathway provides a resource for examining gene functions and broader genomic interactions. We employed the GO plot package together with the clusterProfiler tool in R to assess GO terms and KEGG pathways, thereby gaining deeper insight into the roles of these hub genes (14). Annotation terms with a P value below 0.05 were considered significantly enriched, and the final results were illustrated using bubble diagrams and heatmaps.

### 2.4 Protein-protein interaction network analysis

We constructed a protein-protein interaction (PPI) network to explore potential interactions among the identified differentially expressed genes (DEGs), using data from the STRING database (https://cn.string-db.org) (15). This database offers detailed insights into various forms of interactions, including direct physical associations or indirect regulatory mechanisms involving shared signaling pathways. Only interactions surpassing a combined confidence threshold score of 0.4 were retained for subsequent analysis. Cytoscape software (http://www.cytoscape.org) was employed to visualize the constructed PPI network clearly and intuitively.

### 2.5 Selection and functional analysis of hub genes

Highly interconnected hub genes were identified utilizing the cytoHubba plugin within the Cytoscape software. The selection parameters applied included K-core = 2, degree threshold = 2, maximum depth = 100, and node score threshold = 0.2. To further investigate functional relationships, GeneMANIA (http://www.genemania.org) was employed to establish a protein-protein interaction (PPI) network, facilitating the prediction of gene functions and the identification of genes with similar biological roles. This platform integrates various bioinformatics approaches, including physical interactions, co-expression patterns, co-localization, gene enrichment analysis, genetic interactions, and site prediction. Subsequently, a co-expression network of the selected hub genes was constructed using GeneMANIA, offering a comprehensive framework for uncovering internal gene associations within the dataset (16).

### 2.6 Machine learning and identification of hub genes

To identify key genes associated with gastric cancer (GC)-related membranous nephropathy (MN), we implemented two well-established machine learning approaches: least absolute shrinkage and selection operator (LASSO) regression and random forest. The glmnet and randomForest R packages were utilized to develop predictive models (17–19). For LASSO regression, a three-fold cross-validation strategy was applied to optimize the lambda parameter. We selected the λ.1se (0.05) to avoid overfitting, which provided a more parsimonious model while retaining predictive accuracy. Meanwhile, the random forest algorithm was configured with 1000 decision trees and 50 perturbations to ensure robust feature selection. Genes with a MeanDecreaseGini value ≥0.05 were defined as significant feature genes. In the final step, genes identified by both methods were cross-compared, and those consistently selected were designated as the core GC-associated MN genes.

### 2.7 Receiver operating characteristic curve analysis

To evaluate the diagnostic significance of key genes in membranous nephropathy (MN) datasets (GSE108109), receiver operating characteristic (ROC) analysis was conducted using the ROC function in R. The area under the curve (AUC) was calculated to validate the predictive performance of these genes. Additionally, in subsequent analyses, the clusterProfiler package was employed to perform Gene Set Enrichment Analysis (GSEA) on the finalized hub genes, providing insights into their functional roles and associated biological pathways.

### 2.8 Immune infiltration analysis and correlation with hub gene

To analyze immune cell infiltration patterns in gastric cancer (GC) and membranous nephropathy (MN), the CIBERSORT algorithm was applied to estimate the relative proportions of 22 distinct immune cell types. The vioplot package in R was then used to generate visual representations of these distributions. Subsequently, a correlation matrix was constructed to illustrate the relationships between the immune cell subsets. To further examine the association between hub gene expression and immune cell infiltration, Spearman correlation analysis was conducted, providing insights into potential immune regulatory mechanisms.

### 2.9 Evaluation of Hub genes in relation to disease

The comparative toxicogenomics database (CTD) (http://ctdbase.org/) was used to identify gene-disease interactions (20), the relationship between hub genes and kidney diseases and gastric tumors were analyzed by the CTD database.

### 2.10 Construction of hub genes-targeted drugs and the ceRNA network

The Drug-Gene Interaction Database (DGIdb) was employed to predict drug targets associated with the identified hub genes. We utilized the miRanda, TargetScan, and miRDB databases to predict mRNA-miRNA interaction pairs based on the four identified hub genes. The results common to all three databases were selected for further analysis. Subsequently, we searched for the predicted miRNAs in the Spongescan database and filtered for miRNA-lncRNA pairs, thereby constructing a ceRNA network comprising mRNA-miRNA-lncRNA interactions.

### 2.11 Experimental validation

Through a single-center retrospective analysis, we identified 3 patients with biopsy-confirmed gastric cancer-associated MN in the Nephrology Department of China-Japan Friendship Hospital from 2014-2024. One patient found gastric cancer with lymph node metastasis at the same time as the diagnosis of membranous nephropathy. Another patient found a recurrence of gastric cancer 8 months after diagnosis of MN. The last patient was found with low differentiated gastric adenocarcinoma 2 years after diagnosis of MN. Immunohistochemistry was used to assess the differences in gene expression of gastric cancer-associated MN and primary membranous nephropathy. The samples from 3 gastric cancer-associated MN patients included both kidney tissue and gastric cancer tissue, and IHC staining was performed on both types of tissues. Additionally, 3 PMN patient samples underwent kidney pathology staining. We also performed IHC staining on kidney tissues from 3 healthy controls and gastric tissues from 3 healthy controls in order to compare gene expression differences between kidney tissue and gastric tissue. The baseline characteristics of the three GC-MN patients are as follows: Patient 1 was a 74-year-old male with gastric adenocarcinoma and lymph node metastasis, diagnosed with MN simultaneously (MN stage II, GC stage T3N2M0, received surgical resection and chemotherapy of GC). Patient 2 was a 63-year-old female who developed gastric adenocarcinoma eight months after being diagnosed with MN (MN stage III, GC stage T2N1M0, received surgical resection). Patient 3 was a 31-year-female with poorly differentiated gastric adenocarcinoma diagnosed two years after MN (MN stage II, GC stage T3N3bM1, received chemotherapy and immunotherapy). Control sample selection was based on strict exclusion criteria: primary MN patients were excluded if they had a history of malignancy; healthy controls were excluded if they had any history of kidney disease or malignancy.

Immunohistochemistry staining for *CCND1*, *COL10A1, CEBPD, and BMP2* was performed for 3 gastric cancer-associated MN patients and 3 PMN patients of renal biopsy. Positive and negative controls were used to validate the antibody. The tissue was fixed and embedded in paraffin and sectioned at a thickness of 3 μm. After dewaxing through a hydration process of xylene treatment followed by alcohol baths, the kidney tissue was repaired using an antigen. Antigen repair was performed using high-pressure thermal repair with 2% Ethylene Diamine Tetraacetic Acid (EDTA) buffer for 3 minutes, and endogenous peroxidase was inactivated using hydrogen peroxide peroxidase. The following antibodies (1) *CCND1* antibody (Cell Signaling Technology) diluted 1:50, (2) *COL10A1* antibody (26984-1-AP, Proteintech) diluted 1:50, (3) *CEBPD* antibody (AF9027, affinity) diluted 1:50, (4) *BMP2* antibody (AF5163, affinity) diluted 1:50 were incubated overnight at 4°C, washed with phosphate buffer saline (PBS) obtained from Thermo Fisher Scientific, and then incubated at 4°C for 2 hours. C, washed with PBS and stained with horseradish peroxidase and diaminobenzidine to visualize the reaction. Sections were observed under a bright-field microscope (Nikon, Tokyo, Japan) at a magnification of 400 using a Moticam 2506 instrument (Motic, Fujian, China), and images were taken with a digital camera system (Nikon) for assessment in a blinded manner. Blinding was performed by having independent pathologists, who were not involved in the study, assess the slides without knowledge of the sample identities. Semi-quantitative assessments were performed using Image Pro-plus computer image analysis software (Media Cybernetics, Bethesda, MD, USA) to analyze the average optical density (AOD) and quantify protein levels. Five glomeruli from each biopsy tissue were randomly captured and analyzed, using the average AOD as the final result for each patient.

### 2.12 Statistical analysis

All statistical analyses and visualizations were conducted using R software (version 4.1.2). The packages ggbeeswarm, ggpubr, and ggplot2 facilitated boxplot visualization. Student’s t-test was employed for comparing normally distributed data, whereas Mann-Whitney U tests were applied to data lacking normal distribution. Receiver operating characteristic (ROC) curves and corresponding area under the curve (AUC) values were generated using the pROC package. Correlation networks were visualized through the igraph package, with Spearman’s correlation coefficient utilized to evaluate relationships between continuous variables. Statistical significance was defined as a p<0.05. In the experimental part, for continuous variables with more than two groups, if the data meet the assumption of homogeneity of variances, one-way analysis of variance (ANOVA) was used to compare the differences between the groups. For significant differences found in the ANOVA analysis, Tukey’s *post hoc* test was applied to further compare specific differences between the groups. Statistical significance was defined as a p<0.05.

## 3 Results

### 3.1 Identification of DEGs

**Figure 2:**
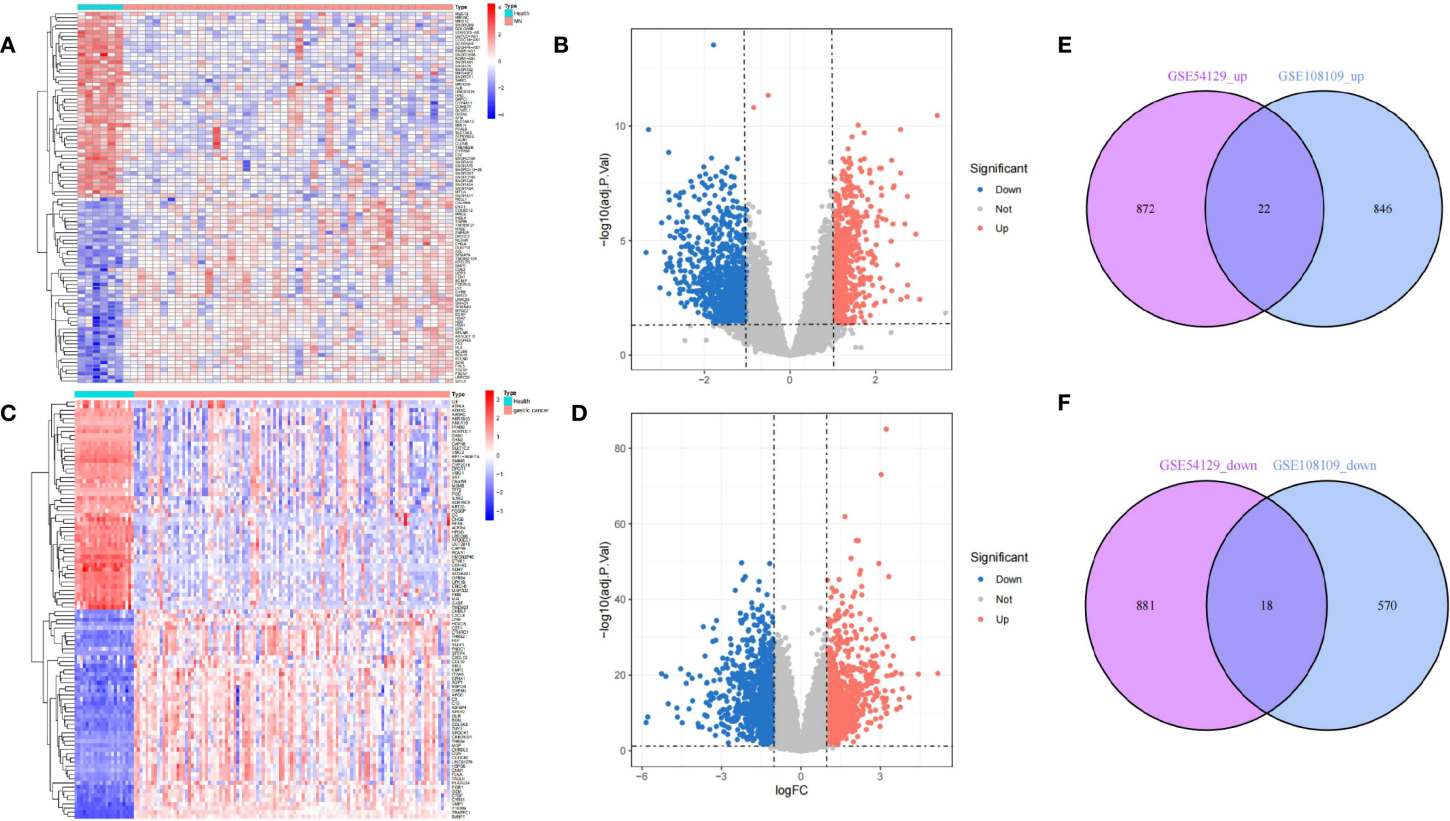
Differentially expressed gene identification. **(A, B)** from GSE108109, **(C, D)** from GSE54129. The volcano plots **(B, D)** show that 1456 and 1793 DEGs were identified from the two datasets, and the heatmaps **(A, C)** show the top 50 up and downregulated genes, respectively. Upregulated genes are in light red; downregulated genes are in light blue. The Venn diagrams show 22 upregulated and 18 downregulated DEGs of GSE108109 and GSE54129 **(E, F)**.

Following normalization, mean gene expression values remained consistent across all samples. To evaluate variability between groups, principal component analysis (PCA) was conducted, confirming the reliability of the dataset. A thorough analysis of the MN dataset (GSE108109) identified 868 upregulated and 588 downregulated differentially expressed genes (DEGs). In the GC dataset (GSE54129), a total of 894 genes were upregulated, while 899 were downregulated. To visualize these findings, a volcano plot (**Figure 2B**) was generated for the MN dataset, with the top 50 highly expressed and lowly expressed genes displayed in a heatmap (**Figure 2A**). Similarly, the GC dataset’s gene expression patterns were illustrated in **Figures 2C, D**. Notably, 22 DEGs were consistently upregulated in both MN and GC datasets (**Figure 2E**), while 18 shared DEGs exhibited downregulation (**Figure 2F**).

### 3.2 GO and KEGG pathway enrichment of common DEGs

**Figure 3:**
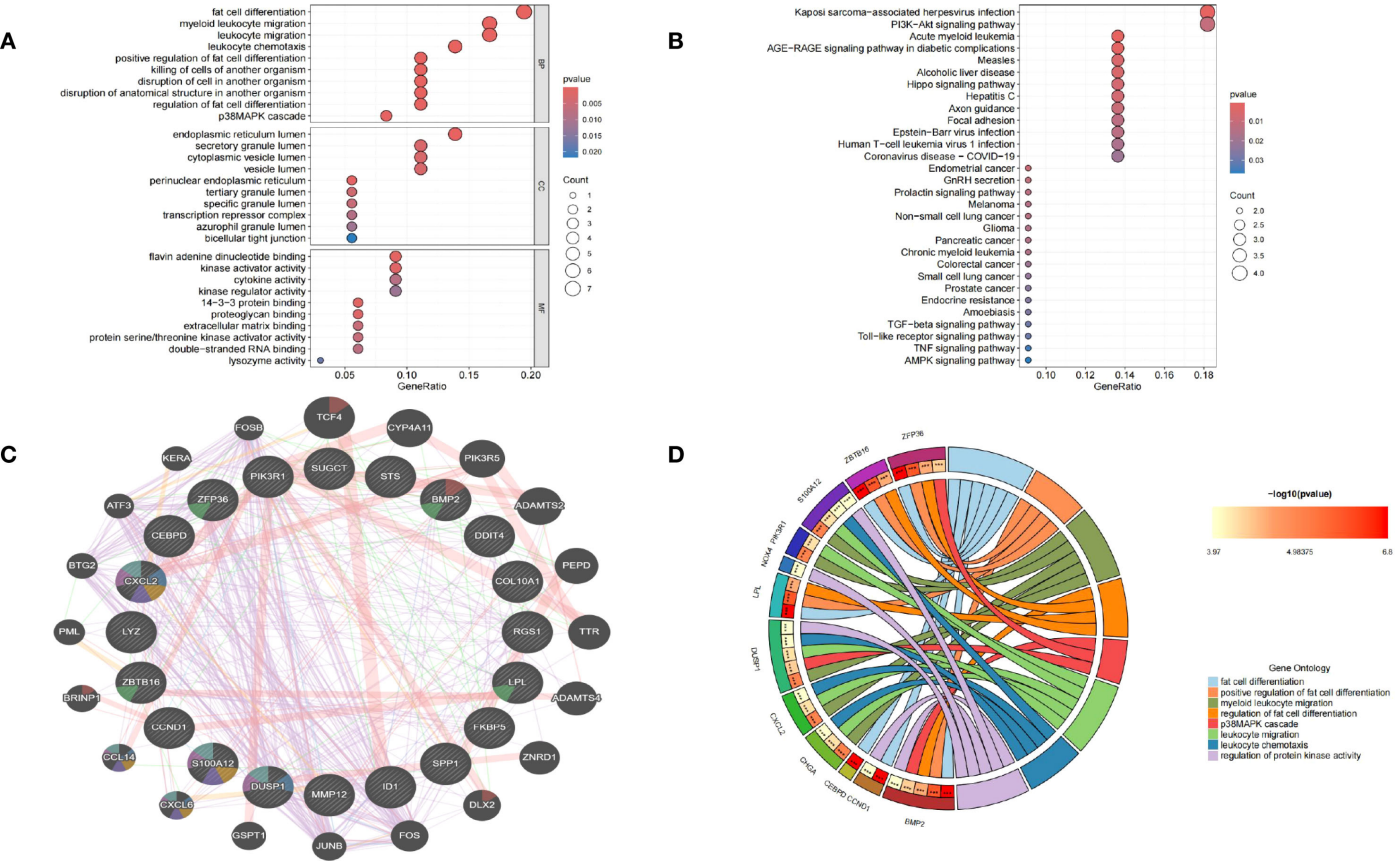
Functional annotation of DEGs and selection of hub genes. **(A)** Bubble chart illustrating the significant enrichment terms of co-expressed DEGs in terms of GO enrichment analysis. **(B)** Bubble chart illustrating the significant enrichment terms of co-expressed DEGs in the KEGG analysis. **(C)** Characterized gene function network of the 20 hub genes. **(D)** GO enrichment analysis of Hub genes.

To elucidate the molecular functions and pathways underlying the connection between membranous nephropathy (MN) and gastric cancer (GC), functional enrichment analyses including Gene Ontology (GO) and Kyoto Encyclopedia of Genes and Genomes (KEGG) were conducted. Results of GO analysis, highlighting the top enriched biological processes (BP), cellular components (CC), and molecular functions (MF), were depicted in bubble charts (**Figure 3A**). Correspondingly, enriched KEGG pathways were illustrated in **Figure 3B**. The subset of biological process (BP) indicated that common DEGs are involved in the fat cell differentiation, myeloid leukocyte migration, leukocyte migration, leukocyte chemotaxis, positive regulation of fat cell differentiation, killing of cells of another organism, disruption of cell in another organism, disruption of anatomical structure in another organism, regulation of fat cell differentiation, p38MAPK cascade. The Cellular Component (CC) subset highlighted involvement in the endoplasmic reticulum lumen, secretory granule lumen, cytoplasmic vesicle lumen and vesicle lumen. Molecular Function (MF) elucidated roles in flavin adenine dinucleotide binding, kinase activator activity, cytokine activity, kinase regulator activity. Furthermore, KEGG pathway enrichment analysis underscored the significant participation of the common DEGs in Kaposi sarcoma−associated herpesvirus infection, PI3K−Akt signaling pathway, Acute myeloid leukemia, AGE−RAGE signaling pathway in diabetic complications, Measles, Alcoholic liver disease, Hippo signaling pathway, Hepatitis C, Axon guidance, Focal adhesion, Epstein−Barr virus infection and Human T−cell leukemia virus 1 infection.

### 3.3 Selection of hub genes between GC and MN

A protein-protein interaction (PPI) network was established using the STRING database and visualized through Cytoscape software. To identify key gene clusters, the MCODE plugin in Cytoscape was applied, setting a combined score threshold of >0.4. The cytoHubba plugin was utilized with the Degree algorithm, leading to the selection of 20 highly interconnected hub genes. A PPI network comprising these 20 genes, represented by 20 nodes and 32 edges, was subsequently constructed and displayed using Cytoscape. Functional annotation of hub genes was conducted through the GeneMANIA database. As shown in **Figure 3C**, these 20 genes were associated with cell chemotaxis, vacuolar lumen response to chemokine, myeloid leukocyte migration, leukocyte chemotaxis and neutrophil migration. GO enrichment analyses were performed on the 20 hub genes to explore the biological functions and pathways involved in MN and GC, GO circle (**Figure 3D**) showed *BMP2, CCND1, CEBPD, LPL, ZBTB16, ZFP36* were involved in fat cell differentiation*. BMP2, ZFP36, ZBTB16, LPL* were involved in positive regulation of fat cell differentiation and regulation of fat cell differentiation. *S100A12, PIK3R1, DUSP1, CXCL2, CHGA* were involved in myeloid leukocyte migration and leukocyte migration. *ZFP36, DUSP1, BMP2* were involved in p38MAPK cascade. *CHGA, CXCL2, DUSP1, S100A12* were involved in leukocyte chemotaxis. *CCND1, BMP2, DUSP1, NOX4, S100A12* were involved in regulation of protein kinase activity.

### 3.4 Machine learning to identify hub GC-associated MN genes

**Figure 4:**
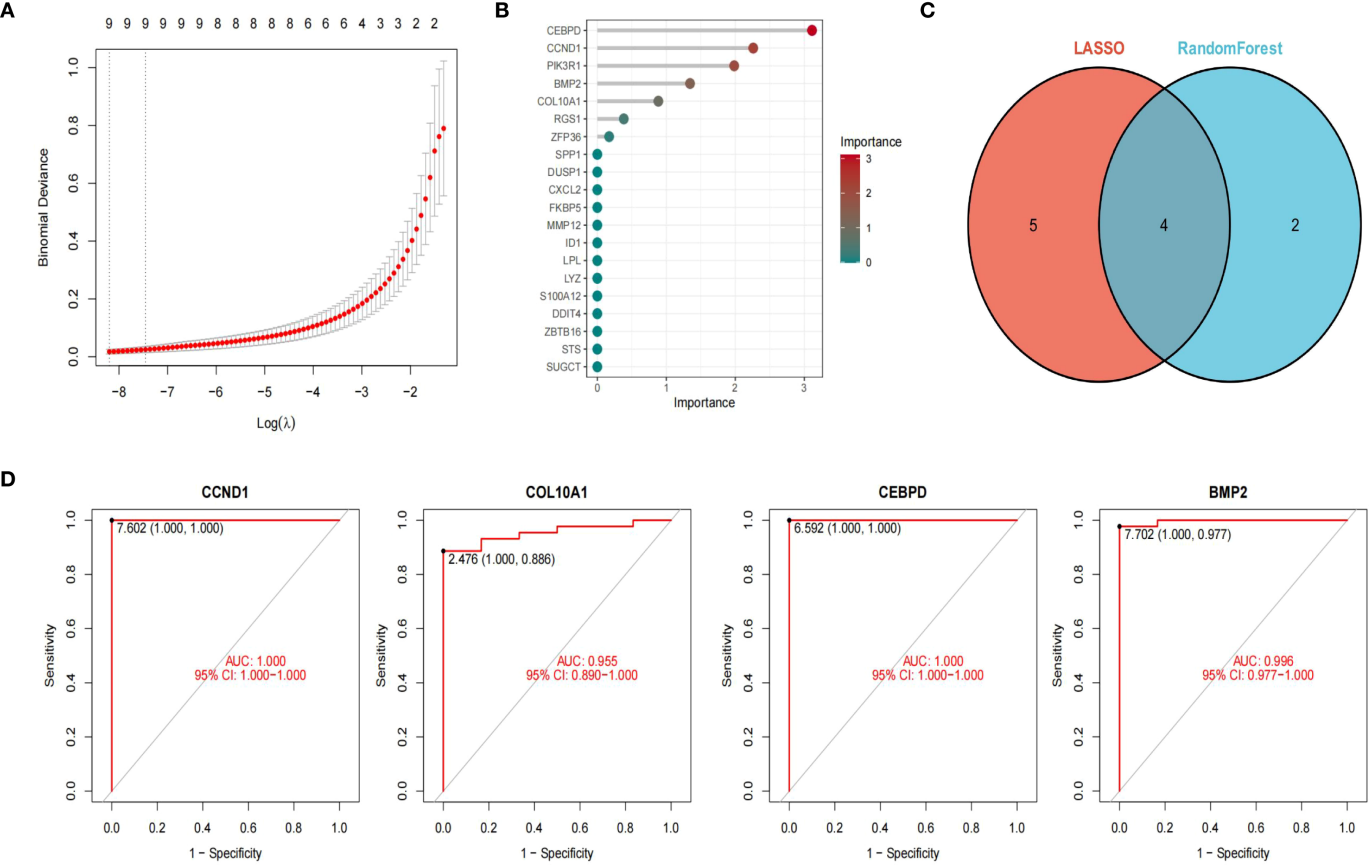
Machine learning to identify hub genes. LASSO algorithm identified 9 significant genes **(A)**. The RFB algorithm confirmed 6 genes **(B)**. Venn diagram of 4 hub genes of GC-associated MN **(C)**. ROC curve showed four genes exhibited satisfactory efficacy in discriminating MN ((*CCND1*: 1.000, *COL10A1*: 0.955, *CEBPD:* 1.000, *BMP2:* 0.996) **(D)**.

We employed two machine learning algorithms to further identify GC-associated MN genes based on the differential analysis of 20 hub genes. The LASSO algorithm, converging on the optimal lambda value, identified 9 significant membranous nephropathy genes (**Figure 4A**). The RandomForest algorithm confirmed 6 membranous nephropathy signature genes (**Figure 4B**). These four genes (*CCND1, CEBPD, COL10A1, BMP2*) were considered by the 2 algorithms to be the hub genes of GC-associated MN (**Figure 4C**). Through ROC analysis, we found that these four genes exhibited satisfactory efficacy in discriminating MN (*CCND1*: 1.000, *COL10A1:* 0.955, *CEBPD:* 1.000, *BMP2:* 0.996) (**Figure 4D**).

For LASSO, λ values were optimized by 10-fold cross-validation, and the λ within 1 standard error of the minimum was selected to avoid overfitting. The cross-validation curve is shown in **Supplementary Figures S1A, B**. For Random Forest, feature importance was evaluated by MeanDecreaseGini index, and stability was assessed across 100 bootstrap resampling runs, with mean ± SD of importance scores reported (**Supplementary Figures S2A, B**).

Survival analysis was conducted using the Kaplan-Meier Plotter online database (https://kmplot.com/analysis/), incorporating clinical information from approximately 875 gastric cancer patients derived from the TCGA dataset. The key hub genes (*CCND1, CEBPD, COL10A1, and BMP2*) were selected as target variables, and patients were stratified into high- and low-expression groups according to the optimal cut-off values. Overall survival (OS) was subsequently analyzed, generating four survival curves. Except for *BMP2*, the survival analyses of the other three genes yielded p< 0.05 and hazard ratios (HRs) > 1, indicating their association with poor prognosis. Among them, *CCND1* emerged as a significant prognostic marker for gastric cancer patients (HR = 1.37, 95% CI = 1.1–1.6; p < 0.001) (**Supplementary Figures S3A–D**).

To further validate the diagnostic efficacy of the identified hub genes, we performed receiver operating characteristic (ROC) curve analysis using two independent datasets, GSE104948 and GSE99339. As shown in **Supplementary Figures S4A, B**, three hub genes *(CCND1, CEBPD*, and *BMP2*) demonstrated favorable diagnostic performance in distinguishing MN samples from controls. In GSE104948, *BMP2 and CEBPD* exhibited the highest diagnostic accuracy, with area under the curve (AUC) values exceeding 0.90, while *CCND1* also achieved robust predictive power (AUC > 0.80). Similarly, in GSE99339, *CCND1, CEBPD*, and *BMP2* consistently maintained strong diagnostic value (AUC > 0.80), whereas *COL10A1* showed only moderate diagnostic performance (AUC < 0.70). These results confirm the reproducibility and robustness of the hub genes across independent cohorts, underscoring their potential as biomarkers for GC-MN.

### 3.5 Association between the hub genes and immune infiltration

**Figure 5:**
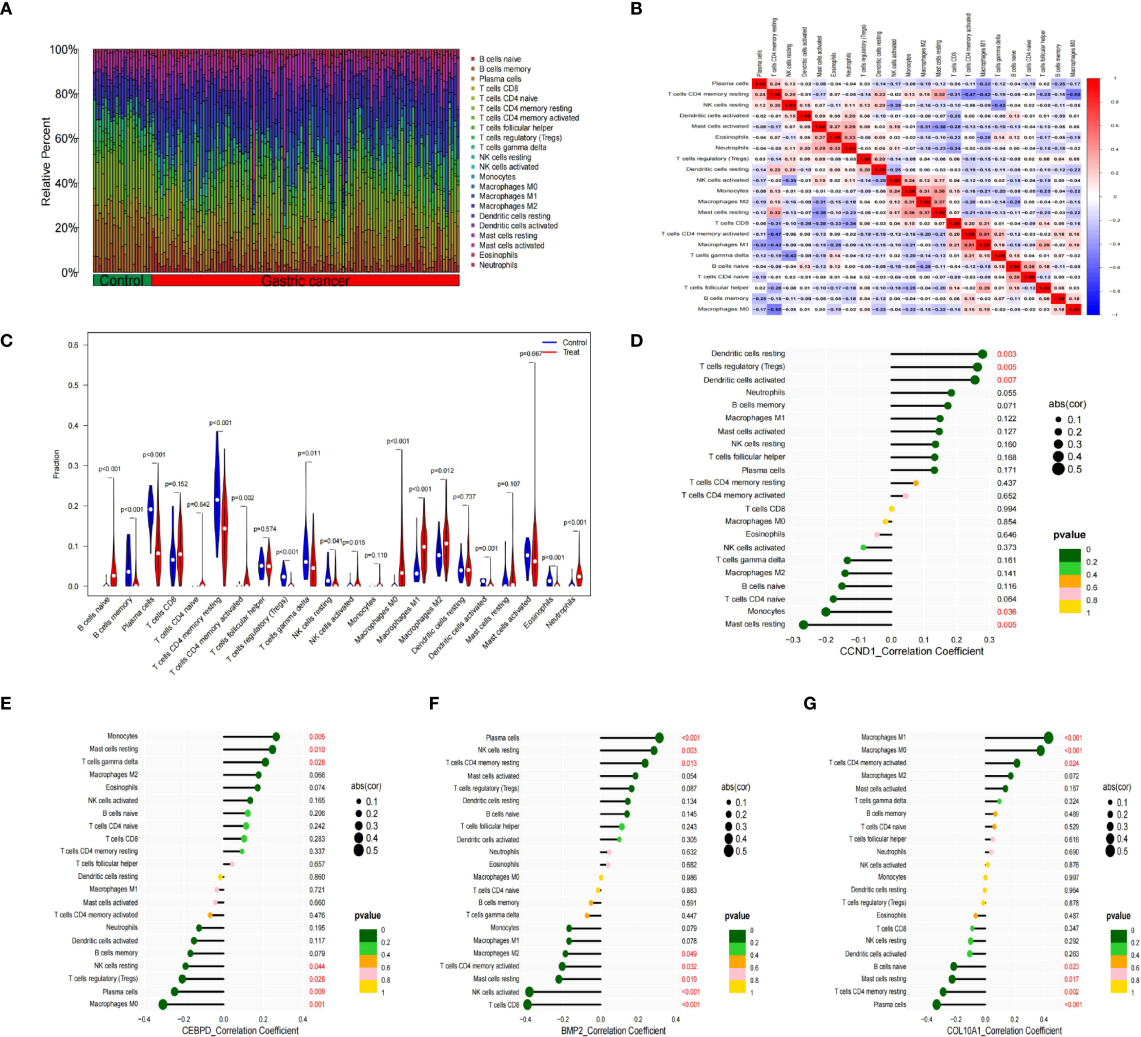
Immune cell infiltration analysis between GC and control. **(A)** The proportion of 22 kinds of immune cells between the two groups. **(B)** Comparison of differential infiltration among 22 immune cells. **(C)** Correlation of 22 immune cell type compositions. **(D-G)** The correlations between the expression of four hub genes (*CCND1, CEBPD, BMP2 and COL10A1*) and immune cell enrichment.

**Figure 6:**
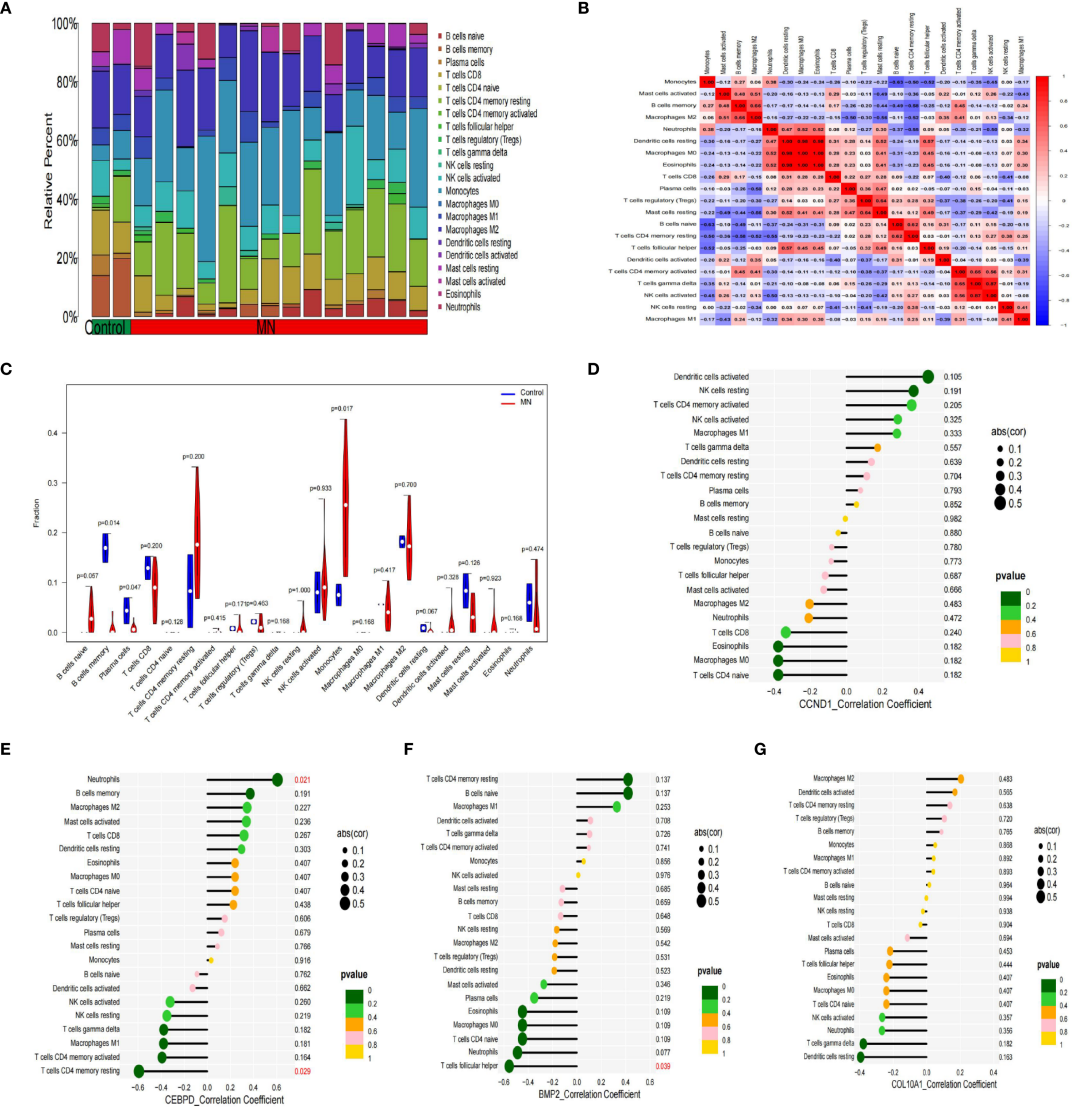
Immune cell infiltration analysis between MN and control. **(A)** The proportion of 22 kinds of immune cells between the two groups. **(B)** Comparison of differential infiltration among 22 immune cells. **(C)** Correlation of 22 immune cell type compositions. **(D-G)** The correlations between the expression of hub genes *(CCND1, CEBPD, BMP2 and COL10A1*) and immune cell enrichment.

Previous studies indicate immune responses and inflammatory processes significantly contribute to membranous nephropathy (MN) and gastric cancer (GC) pathogenesis. To elucidate this immune association, we analyzed the infiltration profiles of 22 immune cell subpopulations using the CIBERSORT algorithm. The differences in immune cell proportions between GC patients and healthy controls are illustrated in (**Figures 5A, B**). Notably, GC samples demonstrated increased proportions of naïve B cells, activated memory CD4+ T cells, activated NK cells, and macrophage subtypes M0, M1, M2, as well as neutrophils, relative to healthy controls. Conversely, memory B cells, plasma cells, resting memory CD4+ T cells, regulatory T cells (Tregs), gamma-delta T cells, activated dendritic cells, and eosinophils were significantly decreased in GC samples. These differential immune cell populations are depicted visually in **Figure 5C**. Spearman correlation coefficients demonstrating associations between immune cell abundance and the expression of the four central genes (*CCND1, CEBPD, COL10A1, and BMP2*) were visualized using lollipop plots (**Figures 5D–G**). Furthermore, differences in immune cell proportions and their correlation networks between MN patients and healthy controls were examined (**Figures 6A, B**). Notably, MN samples showed significantly reduced plasma cells and memory B cells, whereas monocyte infiltration was significantly increased (**Figure 6C**). The associations between immune cell infiltration levels and the four central gene expressions in MN were similarly assessed using Spearman correlation analysis (**Figures 6D–G**).

### 3.6 GSEA analysis

**Figure 7:**
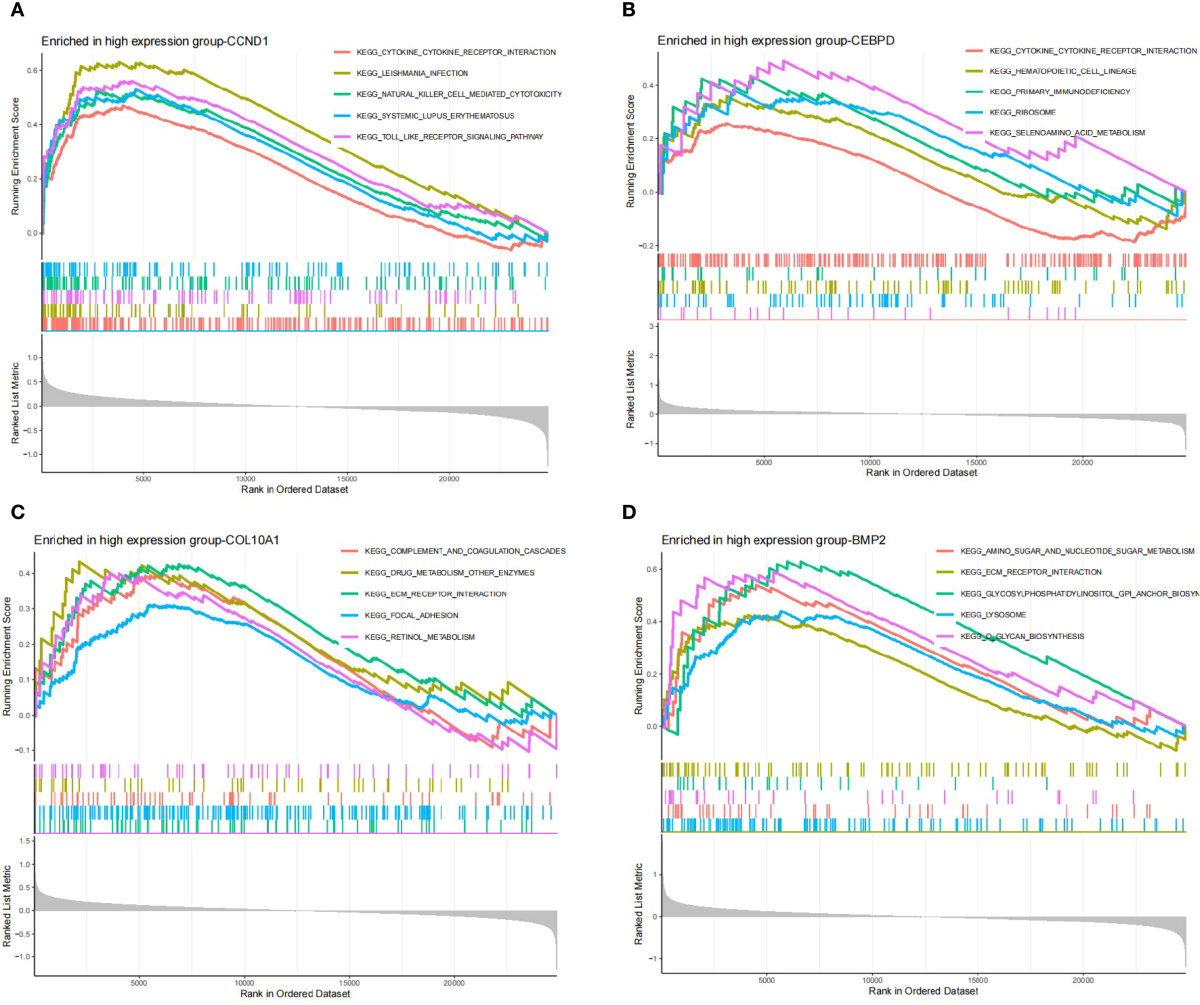
**(A-D)** GSEA analysis of hub genes (*CCND1, CEBPD, COL10A1 and BMP2*).

According to GSEA findings, the *CCND1* high expression group was highly enriched for Cytokine Receptor Interaction, Leishmania Infection, Natural Killer Cell Mediated Cytotoxicity, Systemic Lupus Erythematosus and Toll Like Receptor Signaling Pathway (**Figure 7A**). The *CEBPD* high expression group was mostly concentrated in Cytokine Cytokine Receptor Interaction, Hematopoietic Cell Lineage, Primary Immunodeficiency, Ribosome, Selenoamino Acid Metabolism (**Figure 7B**). Complement And Coagulation Cascades, Drug Metabolism Other Enzymes, Ecm Receptor Interaction, Focal Adhesion, Retinol Metabolism were all associated with increased *COL10A1* expression (**Figure 7C**). The *BMP2* high expression group was mostly concentrated in Amino Sugar And Nucleotide Sugar Metabolism, Ecm Receptor Interaction, Glycosylphosphatidylinositol Gpi Anchor Biosyn, Lysosome, O Glycan Biosynthesis (**Figure 7D**).

### 3.7 Association between hub GC-associated MN genes and disease

**Figure 8:**
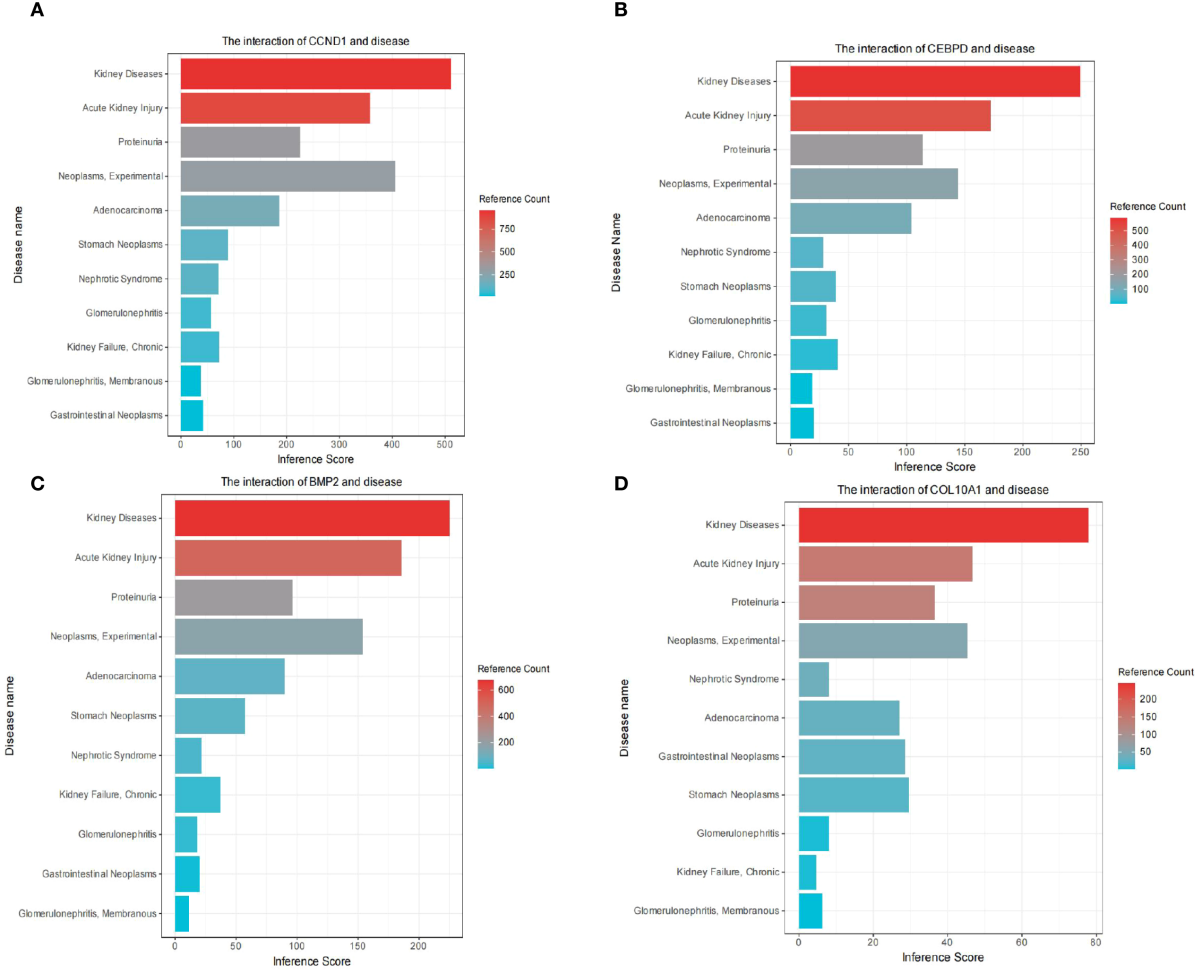
Comparative toxicogenomics database (CTD) analysis. The interaction of *CCND1* and disease **(A)**. The interaction of *CEBPD* and disease **(B)**. The interaction of *BMP2* and disease **(C)**. The interaction of *COL10A1* and disease **(D)**.

We elucidated the association of hub genes with kidney disease and gastric tumors through analysis of the Comparative Toxicogenomics Database (CTD). The results indicated a high correlation of all four hub genes with kidney diseases (**Figures 8A–D**). Moreover, *CCND1, CEBPD, BMP2, COL10A1* exhibited associations with Acute Kidney Injury, Proteinuria, Neoplasms, Adenocarcinoma, Stomach Neoplasms, Nephrotic Syndrome, Chronic Kidney Failure, Gastrointestinal Neoplasms and Glomerulonephritis, Membranous (**Figures 8A–D**).

### 3.8 Prediction of hub genes-targeted drugs

**Figure 9:**
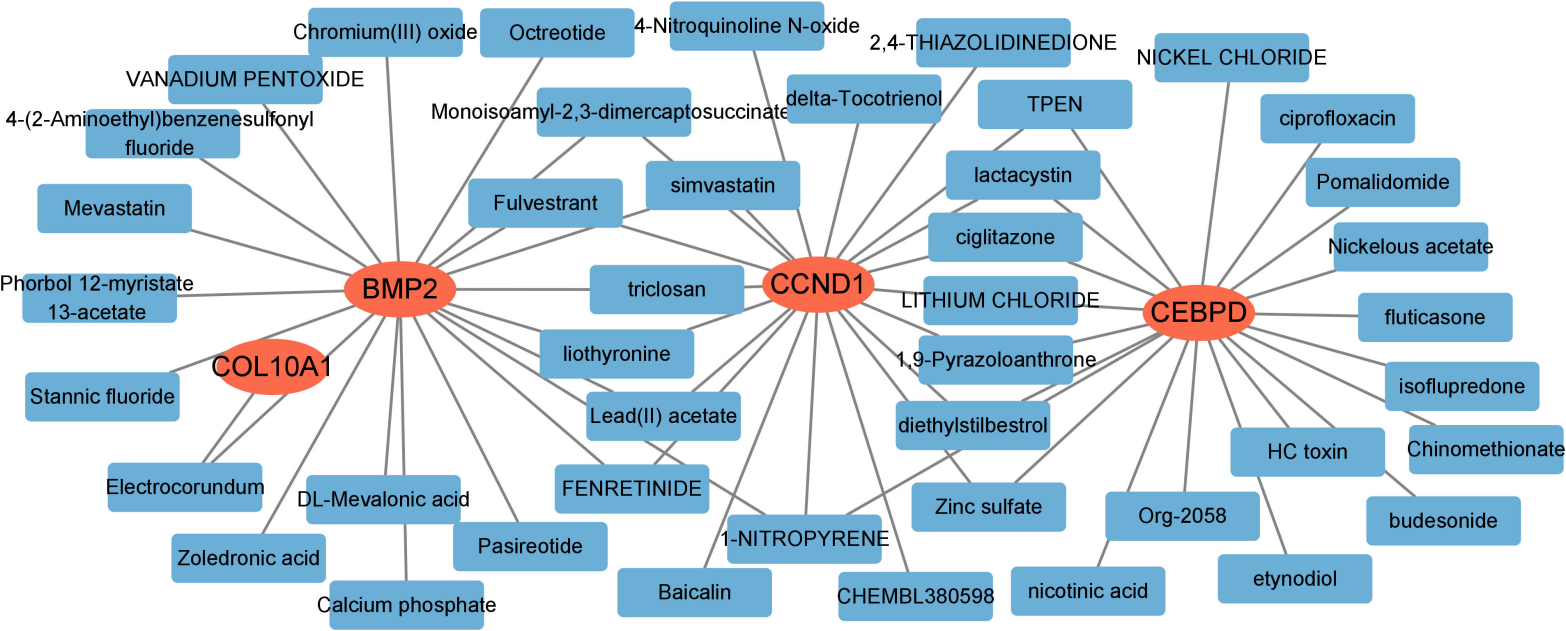
Prediction of targeted drugs for hub genes.

We further investigated potential drugs targeting the hub genes using the DGIdb database and analyzed their interactions, with parameters set to default values. The Cytoscape software was utilized to visualize the 30 targeted drugs for each hub genes (**Figure 9**). A total of 224 drugs targeting the hub genes were identified (**Supplementary Table S1**). Among these, 184 drugs targeted *CCND1*, 24 targeted *CEBPD*, 35 targeted *BMP2* and 1 targeted *COL10A1*.

### 3.9 A ceRNA network based on hub genes

**Figure 10:**
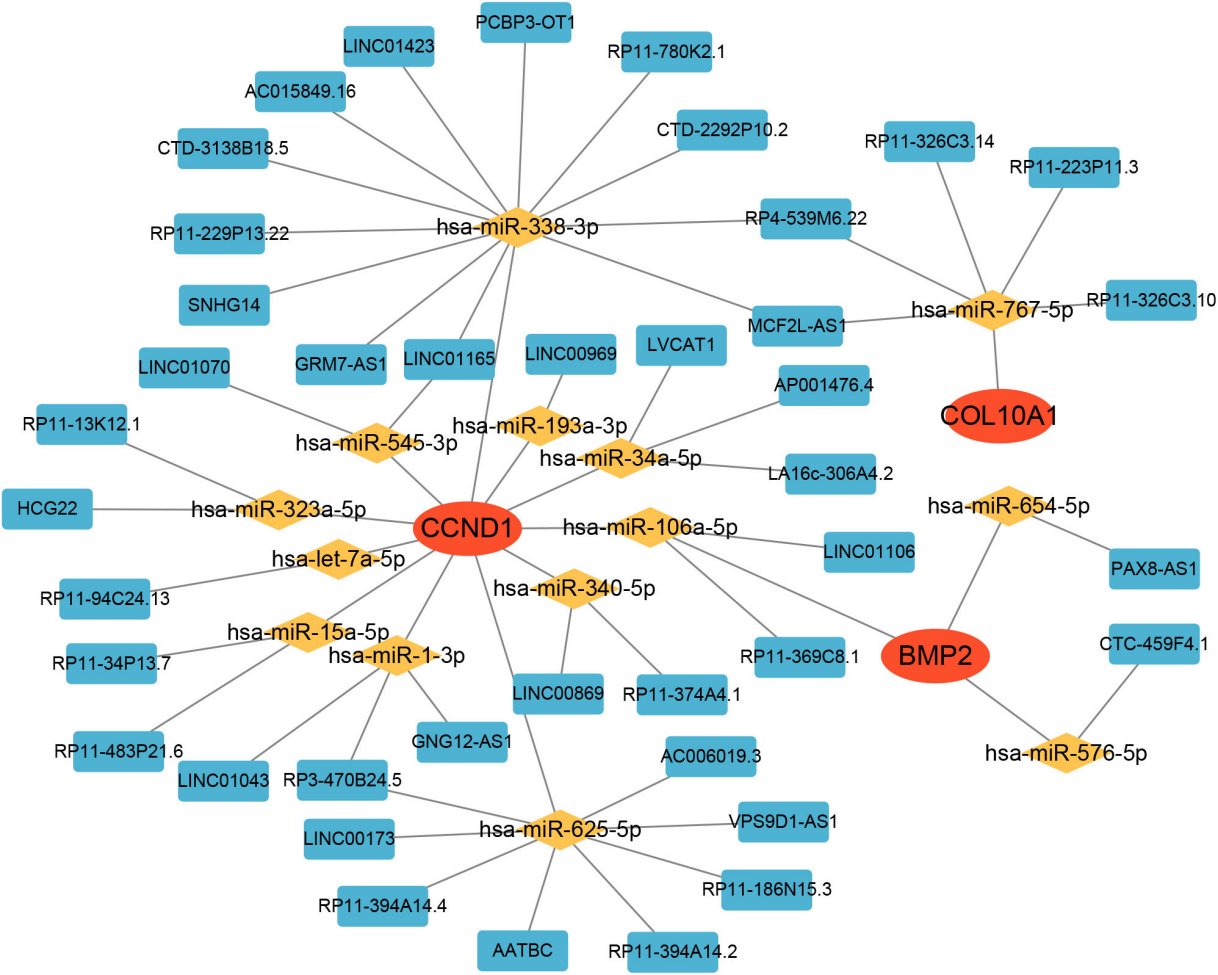
The ceRNA network of hub genes.

Subsequently, we constructed a competing endogenous RNA (ceRNA) network based on the four hub genes using data from the miRanda, TargetScan, miRDB, and Spongescan databases. The resulting network consists of 58 nodes—comprising 3 hub genes, 14 miRNAs, and 41 lncRNAs—and 60 edges (**Figure 10**). The analysis revealed that 36 lncRNAs may regulate the expression of *CCND1* by competitively binding to 11 miRNAs. Additionally, 4 lncRNAs potentially regulate *BMP2* expression through competitive binding with 3 miRNAs, while 5 lncRNAs were found to regulate *COL10A1* expression by targeting a single miRNA. Detailed information on the ceRNA network is provided in **Supplementary Table S2**.

### 3.10 Higher expression of hub genes in gastric cancer-associated MN and tumor tissues

**Figure 11:**
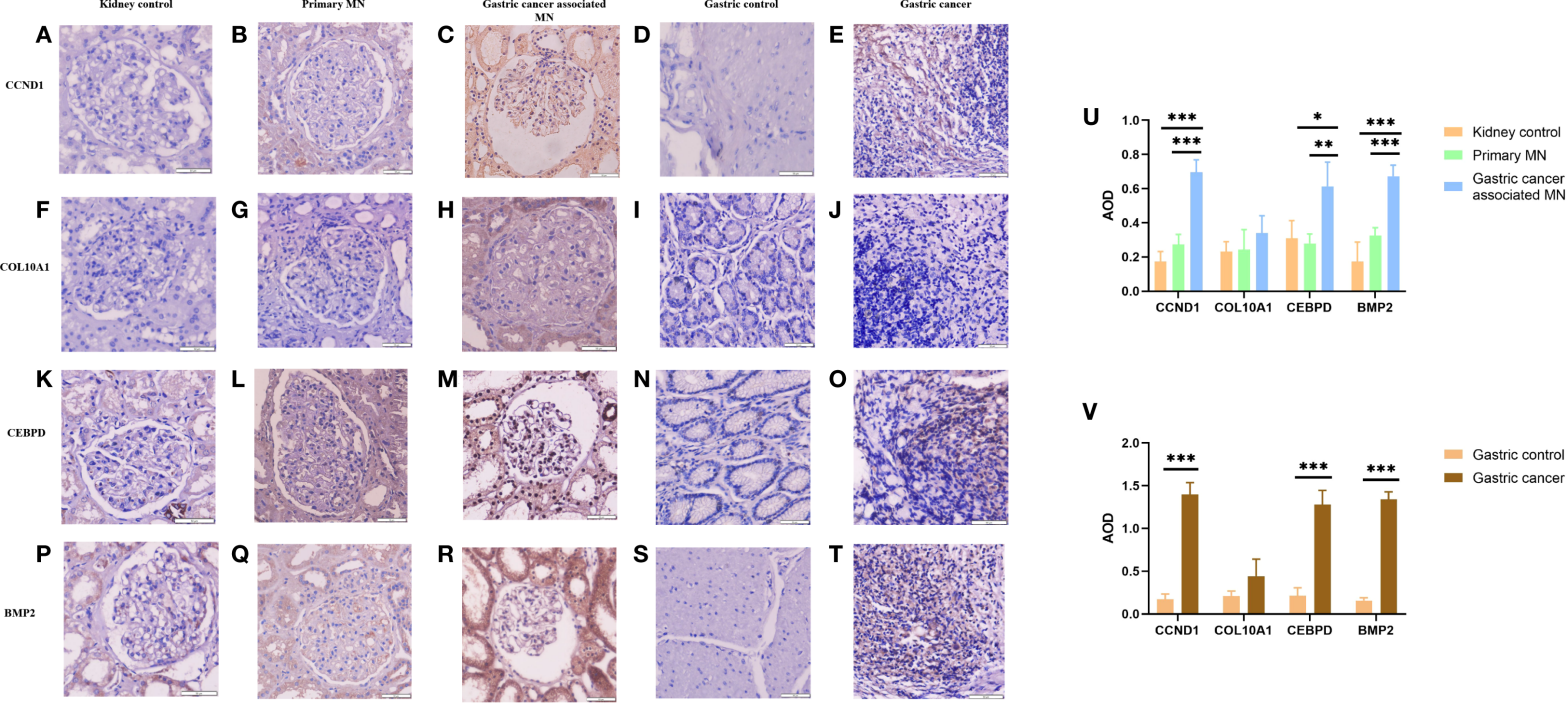
Immunohistochemical expression of CCND1, COL10A1, CEBPD, and BMP2 in renal and gastric tissues **(A-T)**, with quantitative AOD analysis **(U-V)**. *Statistical significance: *p < 0.05, **p < 0.01, **p < 0.001.

Through a single-center retrospective analysis, we identified 3 patients with biopsy-confirmed gastric cancer-associated MN in the Nephrology Department of China-Japan Friendship Hospital from 2014-2023. One patient found gastric cancer with lymph node metastasis at the same time as the diagnosis of membranous nephropathy. Another patient found a recurrence of gastric cancer 8 months after diagnosis of MN. The last patient was found with low differentiated gastric adenocarcinoma 2 years after diagnosis of MN. Immunohistochemistry was used to assess the differences in gene expression of gastric cancer-associated MN and primary membranous nephropathy. We explored the expression difference of hub genes in gastric cancer-associated MN, PMN, and normal kidney tissue, and compared it in gastric cancer and normal gastric tissue by immunohistochemistry. The results showed higher expression of *CCND1*, *CEBPD*, and *BMP2* in gastric cancer-associated MN glomeruli than PMN and normal kidney tissue (**Figures 11A–C, F–H, K–M, P–R**). *CCND1*, *CEBPD*, and *BMP2* were also expressed higher in gastric cancer than normal gastric tissue (**Figures 11D, E, N, O, S, T**).

Immunohistochemistry revealed higher expression of *CCND1, CEBPD, and BMP2* in gastric cancer-associated MN glomeruli compared to PMN and normal kidney tissue (**Figures 11A–C, F–H, K–M, P–R**). Gastric cancer-associated MN glomeruli expressed *CCND1* significantly higher than both normal kidney tissue (0.696 ± 0.073 vs. 0.174 ± 0.058, p=0.001) and PMN (0.696 ± 0.073 vs. 0.274 ± 0.058, p=0.001). Gastric cancer-associated MN glomeruli expressed *CEBPD* significantly higher than normal kidney tissue (0.613 ± 0.142 vs. 0.310 ± 0.104, p=0.013) and PMN (0.613 ± 0.142 vs. 0.279 ± 0.055, p=0.009). Gastric cancer-associated MN glomeruli expressed *BMP2* significantly higher than both normal kidney tissue (0.673 ± 0.045 vs. 0.173 ± 0.114, p=0.001) and PMN (0.673 ± 0.045 vs. 0.325 ± 0.047, p=0.001). *COL10A1* was negative in the glomeruli of gastric cancer-associated MN, PMN, and normal kidney tissue (**Figures 11F–H**). *CCND1* was expressed at higher levels in metastatic lymph nodes from stomach cancer compared to normal gastric tissue (1.396 ± 0.141 vs. 0.174 ± 0.058, p=0.001). Similarly, *CEBPD* (1.280 ± 0.164 vs. 0.212 ± 0.093, p=0.001) and *BMP2* (1.339 ± 0.089 vs. 0.158 ± 0.031, p=0.001) showed higher expression in gastric cancer than in normal gastric tissue (**Figures 11D, E, N, O, S, T**). *COL10A1* expression did not show any significant difference between gastric cancer and normal gastric tissue groups (**Figures 11I, J**). The AOD (Average Optical Density) bar charts for each group are shown in **Figures 11U, V**. (original magnification×400).

## 4 Discussion

The coexistence of MN and malignancies, particularly in middle-aged and elderly individuals, may be coincidental due to overlapping onset ages. However, tumor antigens or tumor-reactive antibodies detected in glomerular immune deposits suggest a pathogenic link (21). Researches have consistently demonstrated a link between MN and various cancers (22) Napat et al. (23) conducted a meta-analysis reporting that approximately 10% of MN cases are linked to cancer. Lung cancer was the most prevalent, followed by gastric, intestinal, prostate, and breast cancers. Despite this, the underlying mechanisms remain unclear. Through biosignature analysis, we identified *CCND1, CEBPD, BMP2, and COL10A1* as key genes in GC-associated MN, with significant diagnostic potential confirmed by ROC analysis. Immunohistochemical validation in GC-associated MN patients showed *CCND1, CEBPD, and BMP2* overexpression, suggesting their role in disease mediation.

### 4.1 Molecular Insights into GC-associated MN

We identified 40 shared DEGs (22 upregulated, 18 downregulated), primarily enriched in pathways related to immune response, inflammation, and cellular processes critical for disease progression. GO and KEGG analyses highlighted key pathways, including leukocyte migration, cytokine activity, and PI3K-Akt signaling, emphasizing the role of inflammatory and immune mechanisms in both MN and GC. The identification of *CCND1, CEBPD, COL10A1*, and *BMP2* as hub genes via machine learning underscores their pivotal roles in GC-associated MN.

*CCND1*, located at 11q13, is frequently amplified in gastric cancer, particularly in the CIN subtype (24). This amplification drives cyclin D1 overexpression, promoting G1/S transition through CDK4/6, thereby accelerating tumor progression (25). Overexpression correlates with poor prognosis, including reduced overall survival and increased recurrence rates, and confers resistance to chemotherapy and radiotherapy, highlighting the need for targeted therapies (26). In podocytes, cyclin D1 plays a key role in maintaining cell cycle quiescence and differentiation. Its dysregulation contributes to glomerulosclerosis by impairing proliferation capacity, leading to incomplete GBM coverage and podocyte injury (27–31).

*CEBPD*, a C/EBP family member, regulates cell proliferation, differentiation, and inflammation. It is significantly downregulated in gastric cancer cells (MKN45, MKN74) compared to normal gastric mucosa, suggesting a tumor-suppressor role (32). In glomerular disease, *CEBPD* regulates SMαA and MCP-1, driving myofibroblast transdifferentiation and inflammatory responses in mesangial cells (33). During acute inflammation, *CEBPD* mitigates renal injury via IL-17 signaling but paradoxically exacerbates fibrosis in later stages (34). Additionally, by upregulating HIF-1α, *CEBPD* protects against hypoxia-induced acute kidney injury through enhanced angiogenesis, antioxidative stress, and metabolic reprogramming (35).

*BMP2*, a TGF-β superfamily member, regulates cell proliferation, apoptosis, and ECM remodeling. In gastric cancer, *BMP2* activates the PI3K/Akt pathway, promoting EMT, invasion, and metastasis, making it a potential therapeutic target (36). Its overexpression correlates with lymph node metastasis, high tumor grade, and poor prognosis (37). Increased serum *BMP2* levels further associate with bone metastasis and tumor burden (38, 39). In the kidney, *BMP2* triggers pSMAD1 signaling, leading to GBM thickening and filtration barrier disruption. Complement activation (C3a, C5b-9) induces *BMP2* secretion, linking immune activation with podocyte injury (40). Additionally, *BMP2* enhances ROS production and Id-1 expression, contributing to cell adhesion dysfunction, ion transport imbalance, and fibrogenesis, key events in membranous nephropathy (41).

*COL10A1*, a collagen family member, is a key mediator of tumor progression and ECM remodeling. Its overexpression in gastric cancer correlates with poor prognosis, advanced tumor stage, and altered immune microenvironment, making it both a biomarker and therapeutic target (42, 43). Elevated plasma and tissue *COL10A1* levels strongly associate with tumor invasion, EMT, and poor clinical outcomes (44, 45). In the kidney, *COL10A1* upregulation marks fibrotic progression in acute kidney injury (AKI) and correlates with poor renal recovery. KLF4-mediated miR-101 upregulation suppresses *COL10A1*, thereby inhibiting EMT and renal fibrosis in ischemia-reperfusion injury (46).

### 4.2 Immune microenvironment and functional pathways

Immune infiltration analysis revealed distinct immune cell patterns in GC and MN. GC showed elevated macrophages and neutrophils, which promote tumor progression via immunosuppressive and pro-inflammatory mechanisms, while MN exhibited increased monocyte infiltration, a hallmark of glomerular inflammation. Strong correlations between hub gene expression and immune cell subtypes (e.g., *CCND1* and *BMP2* with macrophages and neutrophils) further suggest their roles in immune-mediated tissue damage and repair.

Our immune infiltration analysis demonstrated increased macrophages and neutrophils in GC, and enhanced monocyte infiltration in MN, with hub genes *CCND1*, *CEBPD*, and *BMP2* showing significant correlations with these immune subsets. *CCND1* has been shown to regulate T-cell and dendritic cell activity, thereby linking tumor cell proliferation with immune dysregulation. *CEBPD* promotes pro-inflammatory signaling and monocyte/macrophage recruitment, which may exacerbate glomerular injury. *BMP2* is implicated in NK- and B-cell regulation, potentially facilitating immune complex deposition and podocyte damage. These findings suggest that abnormal expression of *CCND1*, *CEBPD*, and *BMP2* synergistically interacts with immune cell infiltration, thereby driving the progression of GC-MN through both tumor-promoting and kidney-injuring mechanisms.

GSEA analysis highlighted functional roles of hub genes in GC-associated MN: *CCND1* was enriched in Toll-like receptor and cytokine receptor pathways, central to innate immune activation and chronic inflammation. *BMP2* was enriched in lysosomal pathways, suggesting its involvement in autophagy and antigen processing, crucial in tumor immunity and glomerular injury.

Functional enrichment highlighted fat cell differentiation and immune cell migration as key pathways. Dysregulated adipogenesis can alter cytokine profiles, contributing to systemic inflammation and immune dysregulation, whereas enhanced immune cell migration may promote renal infiltration and immune complex deposition. These findings provide mechanistic clues on how metabolic-immune interplay may contribute to GC-MN pathogenesis.

### 4.3 Hub genes-targeted drugs and the ceRNA network

In this study, we identified a series of potential therapeutic drugs targeting four hub genes (*CCND1*, *CEBPD, BMP2*, and *COL10A1*) using the DGIdb database. Notably, *CCND1* was associated with the largest number of candidate drugs (n = 184), underscoring its central role as a druggable target in the disease context. As a key regulator of cell cycle progression, *CCND1* dysregulation has been implicated in various malignancies, and the large number of available drugs suggests it may serve as a pivotal therapeutic target in both MN and GC. Conversely, *CEBPD*, *BMP2*, and *COL10A1* were associated with fewer drugs (n = 24, 35, and 1, respectively), which may reflect the relatively limited pharmacological development targeting these genes to date.

To better understand the regulatory mechanisms underlying the expression of these hub genes, we constructed a ceRNA network based on interactions among lncRNAs, miRNAs, and mRNAs. The resulting network revealed a complex layer of post-transcriptional regulation, particularly for *CCND1*, which was regulated by 36 lncRNAs through 11 miRNAs. This finding is consistent with the central role of *CCND1* in the disease network and suggests that its expression is tightly modulated by multiple non-coding RNA elements.

The retrospective analysis of GC-associated MN cases suggests a bidirectional relationship, where malignancy-driven immune dysregulation may trigger glomerular injury, while chronic kidney disease may influence cancer progression. Immunohistochemical validation confirmed significantly higher expression of *CCND1*, *CEBPD*, and *BMP2* in GC-associated MN than in primary MN and normal tissues, reinforcing their potential as diagnostic biomarkers.

Our findings extend previous studies (6, 9) which established molecular mimicry between tumor antigens and podocyte antigens as a key driver of cancer-associated MN. In contrast to well-established MN antigens such as *PLA2R, THSD7A, and NELL-1*, which are podocyte-expressed targets, *CCND1*, *CEBPD*, and *BMP2* are tumor-associated genes that may contribute to MN through systemic immune modulation. This distinction underscores the novelty of our findings, suggesting that GC-MN may represent a paraneoplastic process where tumor-driven immune dysregulation, rather than direct autoantigenicity, initiates glomerular injury.

Unlike *CCND1*, *CEBPD*, and *BMP2, COL10A1* showed negative staining in GC-MN glomeruli despite being identified as a hub gene with strong diagnostic potential. *COL10A1*, a collagen family member, is known to be upregulated in gastric cancer tissues and promotes extracellular matrix remodeling, invasion, and angiogenesis. Its absence in renal glomeruli suggests that *COL10A1* may exert its effects primarily within the gastric tumor microenvironment, indirectly contributing to paraneoplastic renal injury rather than directly mediating glomerular damage. This discrepancy may also reflect post-transcriptional regulation, dataset heterogeneity, or limitations of antibody sensitivity. Therefore, while *COL10A1* remains an informative bioinformatic marker for GC, its clinical relevance for GC-MN requires cautious interpretation and further validation.

### 4.4 Limitations and future directions

While our study provides novel insights into GC-associated MN, certain limitations must be acknowledged. Several limitations should be acknowledged. First, the validation cohort for GC-associated MN was extremely small (n = 3), limiting generalizability. Second, heterogeneity across GEO datasets may introduce bias despite normalization and batch correction. Third, our analysis is associative and cannot prove causality. Fourth, immune infiltration results could be confounded by unmeasured variables such as tumor stage, treatment history, or host background. Although the number of patient samples used for experimental validation in this study is limited, due to the extremely rare clinical cases of gastric cancer-associated MN and the difficulty of obtaining these tissue samples, each sample has high research value. These limitations underscore the need for future large-scale, multi-center, and mechanistic studies to validate and extend our findings.

## 5 Conclusion

In conclusion, this study elucidates the molecular and immunological mechanisms linking MN and GC, identifying *CCND1*, *CEBPD*, and *BMP2* as key genes of disease pathogenesis. The integration of bioinformatics and machine learning provides a comprehensive framework for understanding GC-associated MN, offering new directions for biomarker discovery and therapeutic interventions. Further experimental studies and clinical trials are warranted to validate these findings and explore the therapeutic potential of these hub genes.

## Data Availability

The datasets presented in this study can be found in online repositories. The names of the repository/repositories and accession number(s) can be found in the article/**Supplementary Material**.
