# Supplementary material for: Identification and validation of biomarkers in gastric cancer-associated membranous nephropathy: Insights from comprehensive bioinformatics analysis and machine learning
Source: Front Immunol. 2025 Oct 8;16:1630836. doi: 10.3389/fimmu.2025.1630836 (PMC12540328; doi:10.3389/fimmu.2025.1630836)
Supplement: Supplementary file 2 [file Supplementaryfile1.docx]

Supplementary Materials

# Supplementary Figures


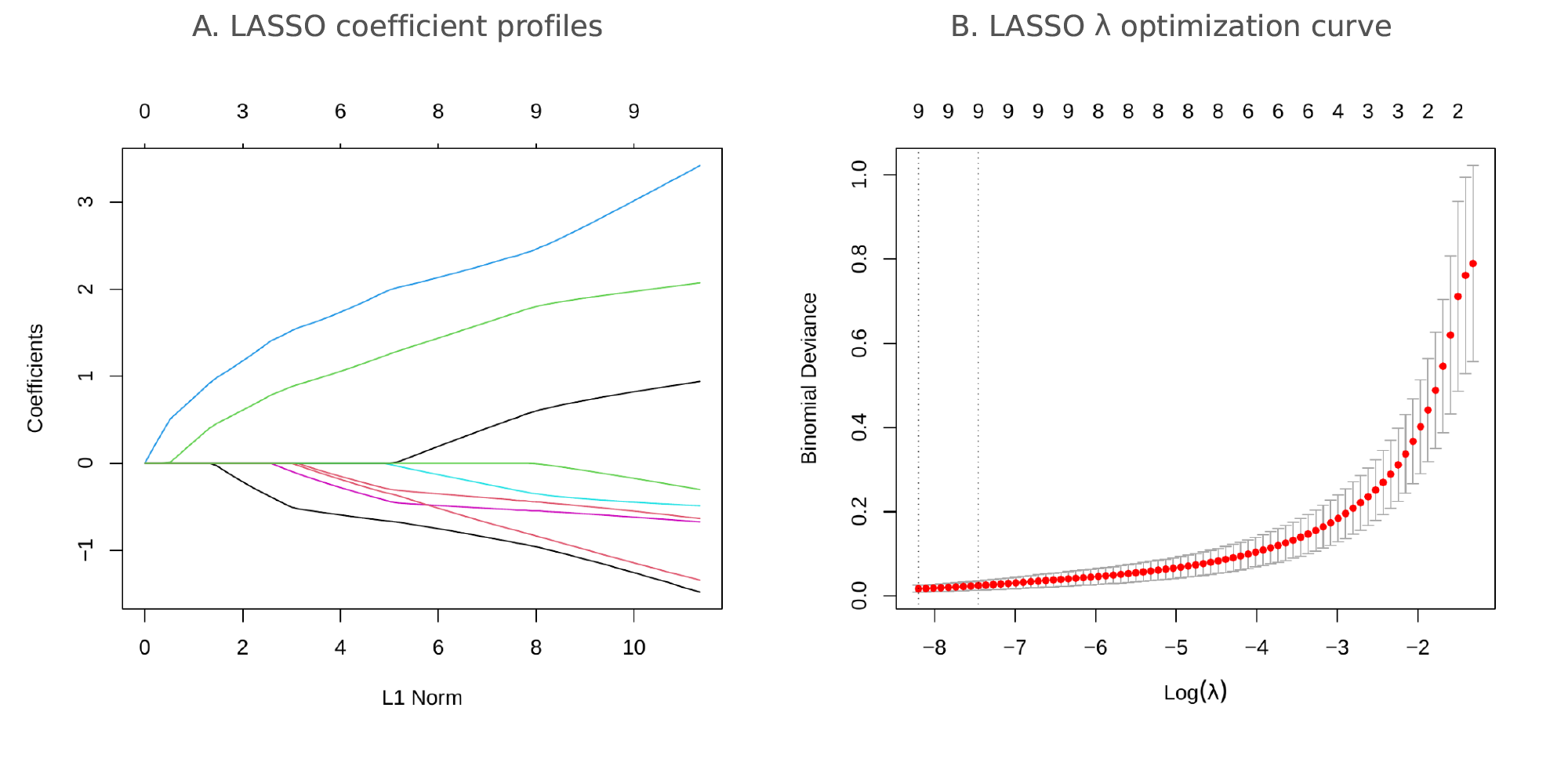


# Supplementary Figure 1. LASSO regression diagnostics. (A) Coefficient profile plot of genes against the L1 norm. As the penalty parameter λ increases, most coefficients shrink toward zero, leaving only a subset of genes with non-zero coefficients.(B) Ten-fold cross-validation curve for LASSO regression. The horizontal axis indicates log(λ), and the vertical axis shows the binomial deviance with standard error bars.


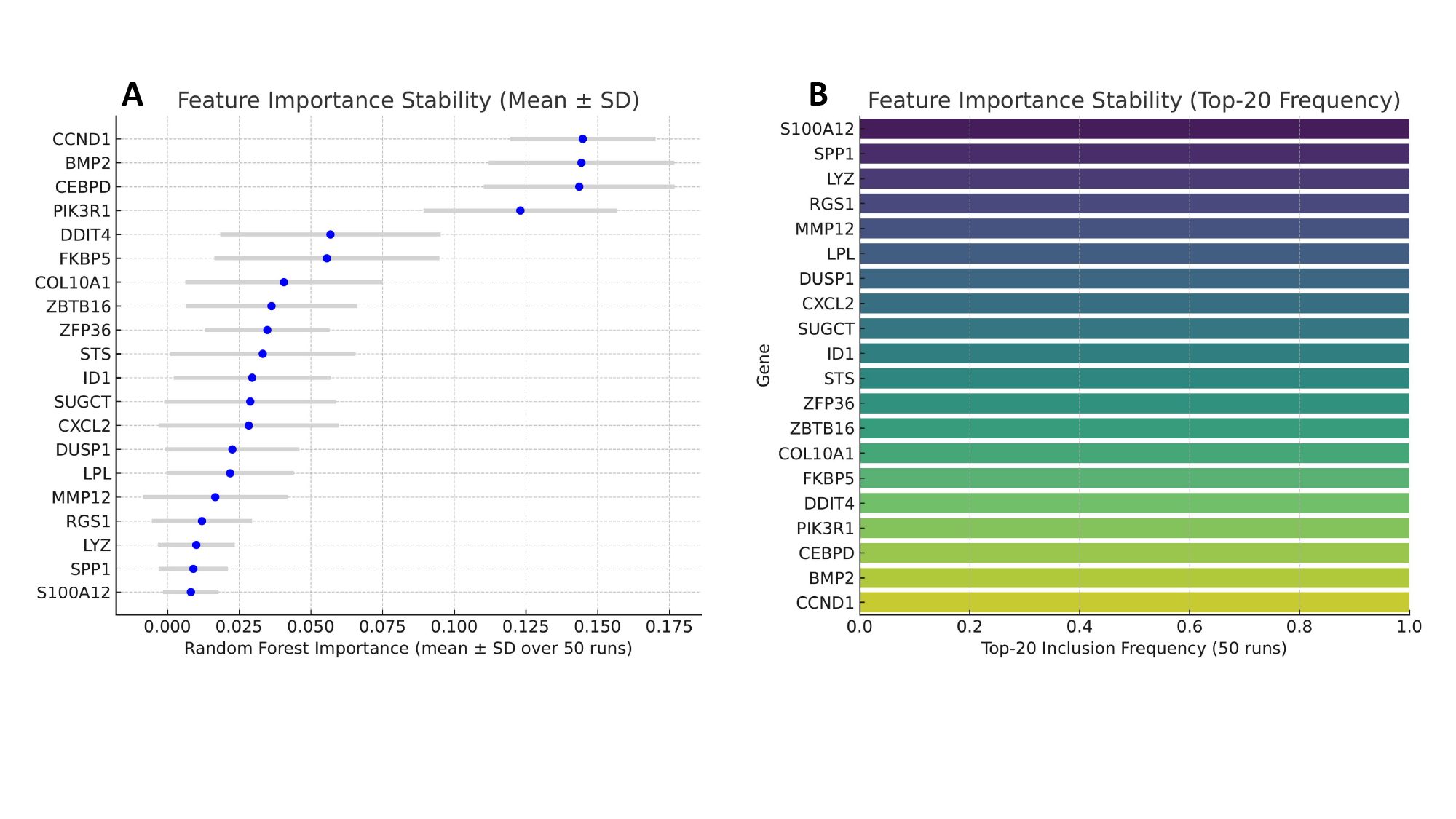


# Supplementary Figure 2. Random forest feature importance stability validation. (A) Mean ± SD of permutation importance (MeanDecreaseAccuracy) for the top 20 genes across 100 bootstrap runs. Error bars indicate across-run variability, reflecting stability of feature selection.(B) Frequency with which each gene was included among the top 20 across 100 runs. Genes such as CCND1, CEBPD, BMP2, and COL10A1 consistently ranked highly, demonstrating robust selection stability.


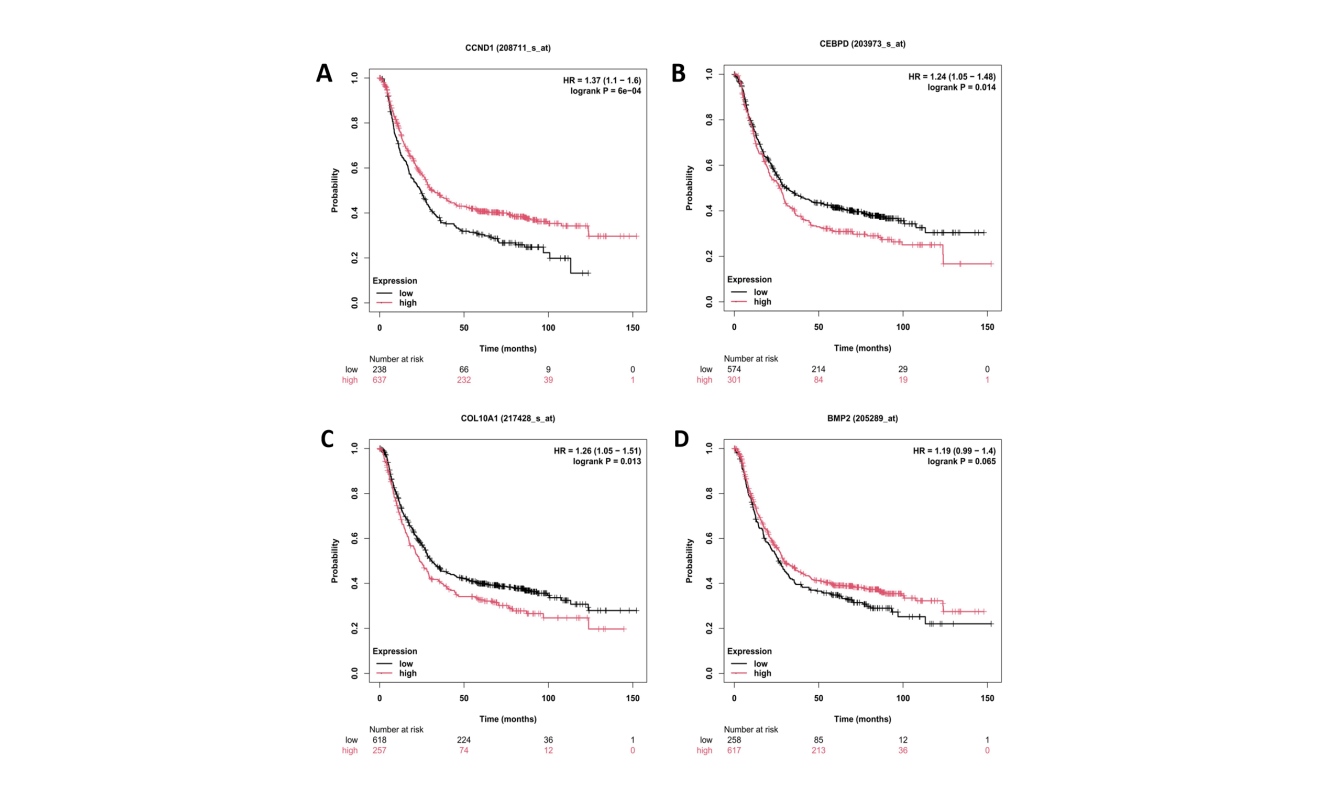


# **Supplementary Figure 3.** Survival analysis in the TCGA-GC cohort. Kaplan–Meier survival curves showing that higher expression of CCND1 (A), CEBPD (B), COL10A1 (C), and BMP2 (D) was significantly associated with worse prognosis in gastric cancer patients from the TCGA-GC cohort.


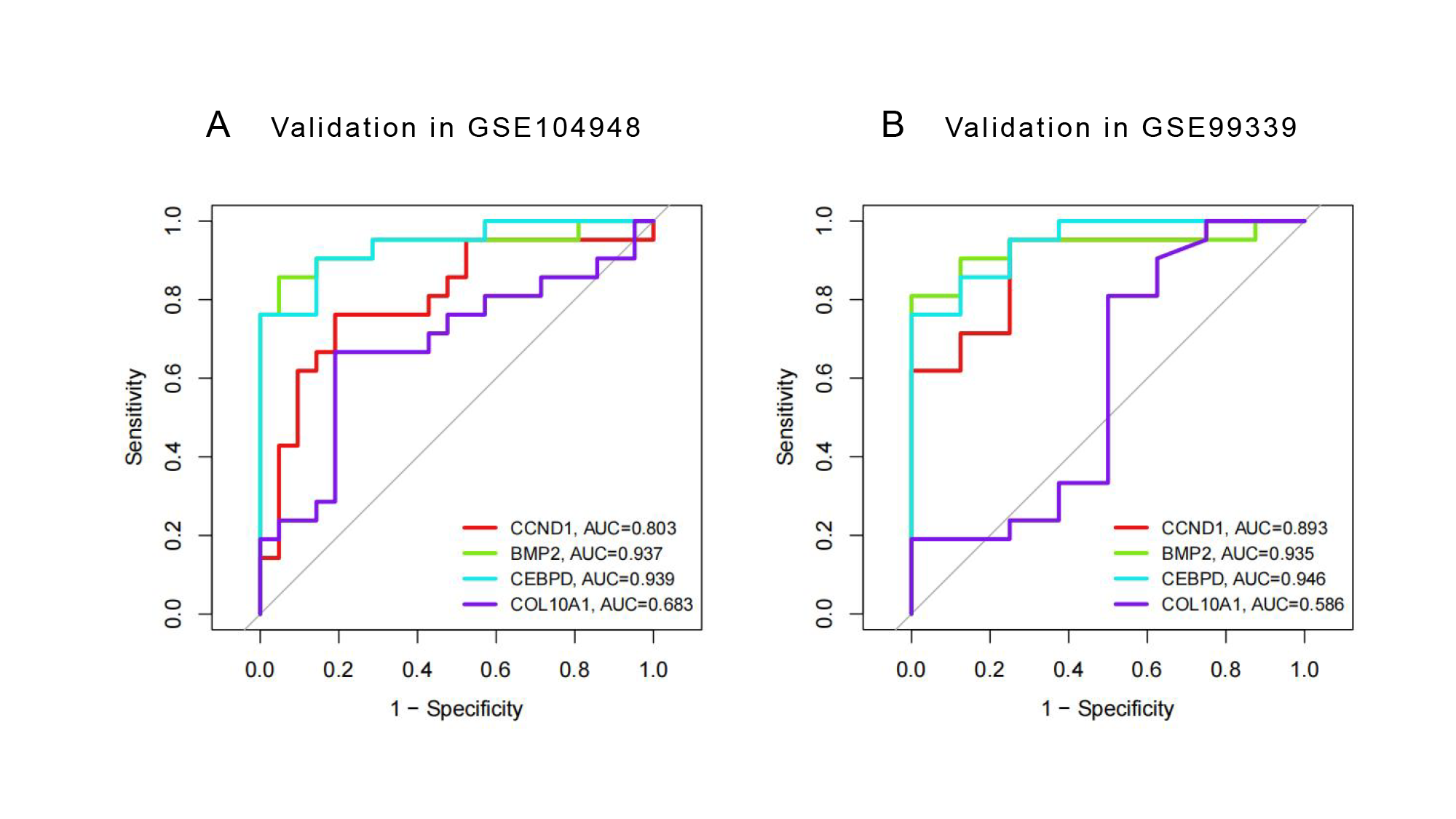


**Supplementary Figure 4.** ROC curve analysis of the four genes (CCND1, CEBPD, BMP2, and COL10A1) in discriminating membranous nephropathy from controls.
(A) Validation in GSE104948 dataset. (B) Validation in GSE99339 dataset.
